# Bioenergetic Strategy for the Biodegradation of p-Cresol by the Unicellular Green Alga *Scenedesmus obliquus*

**DOI:** 10.1371/journal.pone.0051852

**Published:** 2012-12-14

**Authors:** Aikaterini Papazi, Konstantinos Assimakopoulos, Kiriakos Kotzabasis

**Affiliations:** Department of Biology, University of Crete, Heraklion, Crete, Greece; US Dept. of Agriculture – Agricultural Research Service (USDA-ARS), United States of America

## Abstract

Cultures from the unicellular green alga *Scenedesmus obliquus* biodegrade the toxic p-cresol (4-methylphenol) and use it as alternative carbon/energy source. The biodegradation procedure of p-cresol seems to be a two-step process. HPLC analyses indicate that the split of the methyl group (first step) that is possibly converted to methanol (increased methanol concentration in the growth medium), leading, according to our previous work, to changes in the molecular structure and function of the photosynthetic apparatus and therefore to microalgal biomass increase. The second step is the fission of the intermediately produced phenol. A higher p-cresol concentration results in a higher p-cresol biodegradation rate and a lower total p-cresol biodegradability. The first biodegradation step seems to be the most decisive for the effectiveness of the process, because methanol offers energy for the further biodegradation reactions. The absence of LHCII from the *Scenedesmus* mutant wt-lhc stopped the methanol effect and significantly reduced the p-cresol biodegradation (only 9%). The present contribution deals with an energy distribution between microalgal growth and p-cresol biodegradation, activated by p-cresol concentration. The simultaneous biomass increase with the detoxification of a toxic phenolic compound (p-cresol) could be a significant biotechnological aspect for further applications.

## Introduction

Phenolic compounds are environmental pollutants because of their widespread use and potential toxicity to higher organisms. p-Cresol (4-methylphenol) is used in disinfectants and fumigants, in the manufacturing of synthetic resins, in photographic developers, and in explosives [1]. It is highly toxic, corrosive, causes nervous system depression, and therefore is listed as a priority pollutant by the U.S. Environmental Protection Agency [2].

There are several reports on the degradation of p-cresol by microorganisms under anaerobic [3] and aerobic conditions [1], [4], [5], through ortho- and meta- cleavage pathways.

Transformation of methylaromatics via the ortho-cleavage pathway often leads to the accumulation of 4-carboxymethyl-methylbut-2-en-1,4-olides [6], [7]. Only a few naturally occurring microorganisms [8], [9], [10] as well as genetically modified organisms [11] can degrade methylaromatics completely via the ortho-cleavage pathway.

Naturally occurring methylphenols are degraded via the meta-cleavage pathway [4]. *Alcaligenes eutrophus* 335 (ATCC 17697) degrades p-cresol via a catechol meta-cleavage pathway [12], [13], whereas *Ochromonas danica* and *Pseudomonas* sp. CP4 catabolize all cresol isomers via the meta-cleavage pathway using them as the sole source of carbon and energy [14].

The idea of using microalgae in bioremoval processes in general was initially proposed by Oswald and Gotaas (1957) [15]. While the mechanisms of biotransformation and biodegradation of xenobiotics have been extensively studied in bacteria [16], [17], [18], higher plants [19] and animals [20], there is a paucity of data on biodegradation and biotransformation of xenobiotics in algae [21], [22], [23], [24], [25], [26]. Nevertheless, the use of microalgae in bioremoval shows several advantages such as: (a) the utilization of a cheap and abundant energy source (sunlight); (b) the production of biomass for animal feed; (c) the production of high added-value compounds and fine chemicals [27].

Additionally, the biodegradation of phenolic compounds by microalgae seems to be not a simple feature of a particular organism, as it was thought to be before, but mostly a light dependent bioenergetic process that is affected from the growth conditions, especially from the level of exogenously supplied energy as organic carbon or light for photosynthetic energy production [22]. As a result, microalgal growth conditions are the keys for the manipulation of xenobiotics biodegradation and this is their most significant advantage of using microalgae instead of bacteria or fungi. Safonova et al, (2005) [28] showed that *Scenedesmus obliquus* can biodegrade phenanthrene up to 42% in BBM medium [29], but only 24% in Kuhl medium [30]. Ιn our previous publication [22], we proved that phenol biodegradation was taken place by the microalga *Scenedesmus obliquus* only in the absence of any exogenous carbon supply from the culture medium. The most impressive was that the biodegradation was controlled by the light intensity. The almost 10% of phenol biodegradation in 50–60 µE, was increased to 100% in 100–120 µE, while all the other experimental culture conditions were exactly the same. Later, Ke et al, (2010) [31] proved that the freshwater green alga *Selenastrum capricornutum* can degrade polycyclic aromatic hydrocarbons (PAHs), if the alga exposure to metal stress.

The type and the position of the substituent in the phenolic ring, the bond dissociation energy, as well as the inductive and resonance effect phenomena of the substituents adjust the biodegradability of the phenolic compounds [22], [23]. In the case of methylphenol biodegradation by microalgae, the further the donor group (OH^−^) of the phenolic compound is from the second donor group (CH_3_^+^), the higher the biodegradation values are (2-methylphenol <3-methylphenol <4-methylphenol) [23].

The present contribution aims to study the bioenergetic strategy of the microalga *Scenedesmus obliquus* to biodegrade p-cresol in various concentrations (0.15 – 2.5 mM). In the present contribution, we particularly studied the changes in the molecular structure and function of the photosynthetic apparatus of the microalga through the p-cresol biodegradation procedure, as well as the energy distribution between microalgal growth and p-cresol biodegradation by different p-cresol concentrations in order to understand the importance of the bioenergetic balance, via cellular energy management.

## Materials and Methods

### Organism and Growth Conditions

Axenic cultures of unicellular green alga *Scenedesmus obliquus*, wild type D3 [32] and the mutant wt-lhc were autotrophically grown in liquid culture medium [33]. The mutant wt-lhc has a functional photosynthetic apparatus like the wild type, but lacks the capability to synthesize chlorophyll b and thus the ability to form a functional LHCII. Axenic mother cultures were cultivated for one week, in controlled temperature (30°C) and light (150 µmol m^−2^ s^−1^) conditions. They were continuously percolated with air for CO_2_ supply and sedimentation avoidance.

Axenic subcultures with an initial concentration of 2 µL packed cell volume (PCV) mL^−1^ were distributed into 100 mL hermitically sealed bottles (diameter 5 cm, height 9,5 cm) with septa for all the experimental procedures. The final culture volume in each bottle was 50 mL. The only carbon sources in the bottles at the beginning of each experiment (except p-cresol) were the cellular carbon stock from the mother cultures, 0.036% (v/v) of CO_2_, which were present in the 50 mL of the bottle’s air volume and 0.01 % (v/v) of methanol used for the p-cresol dilution. The experiments were performed in a temperature-controlled room (30°C) at a light intensity of 50–60 µmol m^−2^ s^−1^. p-Cresol was dissolved in methanol and added in concentrations of 0.15 mM, 0.3 mM, 0.5 mM, 0.75 mM, 1.0 mM, 1.5 mM, 2.0 mM and 2.5 mM. The corresponding methanol amount was also added to the control cultures. The above p-cresol concentrations were tested for their effects on algal cultures during the entire incubation time of 5 days. Sampling was take place daily in the same time in sterile conditions using sterile needles without opening the bottles, because they have septum in the top of the bottle.

All the cultures tested for possible contamination with bacteria and fungi before (mother cultures) and after the treatments (fifth incubation day) microscopically and by streaking and plating on agar medium.

### Fluorescence Induction Measurements

The Handy Plant Efficiency Analyser, PEA (Hansatech Instruments, Kings’s Lynn, Norfolk, UK) was used for the fluorescence induction measurements. The maximum yield of photochemistry (F_v_/F_m_), the functional antenna size per active reaction center (ABS/RC), the dissipation energy per active reaction center (DI_o_/RC) and the density of active photosynthetic reaction centers (RC/CS_o_) were measured according to the JIP method of Strasser and Strasser (1995) [34]. This method is based on the measurement of a fast fluorescence transient with a 10 µs resolution in a time span of 40 µs to 1 s. Fluorescence was measured at 12-bit resolution and excited by three light-emitting diodes providing a saturated light intensity of 3000 µmol m^−2^ s^−1^ of red (650 nm) light. This method allows the dynamic measurement of a photosynthetic sample at a given physiological state.

### Polarographic Measurements

Maximal photosynthetic and respiratory rates were determined polarographically at 30°C with a Clark type electrode system (Hansatech Instruments, Kings’s Lynn, Norfolk, UK) according to the method of Delieu and Walker (1981) [35]. The actinic light (500 µmol m^−2^ s^−1^) was generated with a light source (MILLE LUCE M1000) and its intensity was measured with a sensitive PAR/temperature sensor (Hansatech, Quantitherm). The infrared part of the applied irradiation was filtered off by inserting a 2% CuSO_4_-containing cuvette (4 cm path length) into the light beam. The cell suspension was adjusted before each measurement to 10 µL PCV mL^−1^.

### High-performance Liquid Chromatography (HPLC) Analysis of Phenolic Compounds

For the phenolic compounds analysis, culture samples were centrifuged for 5 min at 1500 g and the supernatants injected into HPLC, according to the isocratic method of Lovell et al. (2002) [36]. The analyses were performed with a Shimadzu Liquid Chromatography apparatus (LC-10AD) equipped with a SPD-M10A diode array detector (Shimadzu SPD-M10A) and a narrow-bore column (C18, 2.1×150 mm, 5 µm particle size hypersil, SUPELCO). The mobile phase was methanol:water:acetic acid (50∶49:1) at a flow rate of 0.2 mL min^−1^. Detection was carried out by measuring absorbance at 280 nm. The quantification of the compounds was based on the absorbances of known quantities of phenolic compounds.

### Determination of Growth

The culture’s growth rate was estimated by measuring the packed cell volume (PCV) of the culture according to the method of Senger and Brinkmann (1986) [37]. The PCV of a cell suspension was determined by centrifugation at 1500 g for 5 min using haematocrite tubes and expressed as µL PCV (mL culture)^−1^.

### Pigment Extraction and Quantitative Estimation

After centrifugation of the culture at 1500 g for 5 min, the algal pellet was exhaustively extracted with hot methanol until it was colorless. The amount of total chlorophyll was estimated photometrically according to the method of Holden (1965) [38].

### Quantitative Determination of Methanol

Determination of the actual methanol concentrations in the culture medium was performed according to the method of Wood and Siddiqui (1971) [39].

### Data Analysis

Each treatment included three independent bottles and two samplings were carried out of each individual bottle. Standard deviations of the average values are presented on diagrams.

## Results and Discussion

In a previous publication, it was established that mono-substituted methylphenols can serve as alternative carbon sources for carbon depleted *Scenedesmus obliquus* cultures [23]. The biodegradability order was 2-methylphenol < 3-methylphenol < 4-methylphenol due to inductive and resonance effects of the methyl group (electron donor). In order to identify the microalgal bioenergetic strategy of the biodegradation of methylphenols, the most biodegradable of them, 4-methylphenol (p-cresol) was tested. A series of increasing p-cresol concentrations (0 mM, 0.15 mM, 0.3 mM, 0.5 mM, 0.75 mM, 1.0 mM, 1.5 mM, 2.0 mM and 2.5 mM) was used in axenic autotrophic cultures of *Scenedesmus obliquus*.

**Figure 1:**
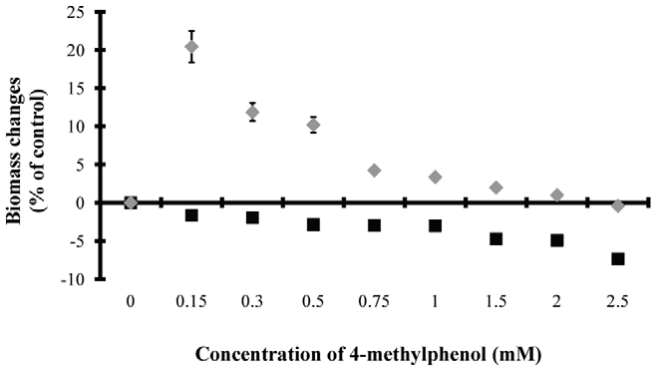
p-Cresol impact on microalgal growth. Impact of increasing 4-methylphenol (p-cresol) concentration on microalgal biomass changes after 24 hours (*black square*) and 5 days incubation (*grey diamond*).

The impact of the increasing p-cresol concentration on microalgal growth is presented in Fig. 1. It was observed that, after 24 hours adaptation, higher p-cresol concentration resulted in higher growth inhibition. However, after five days incubation, two distinct groups emerged. The treatments with concentrations 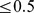 mM led to increased growth rates (10–20 % of control), while the concentrations 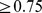 mM led to growth rates similar to the one determined for control treatments (Fig. 1).

**Figure 2:**
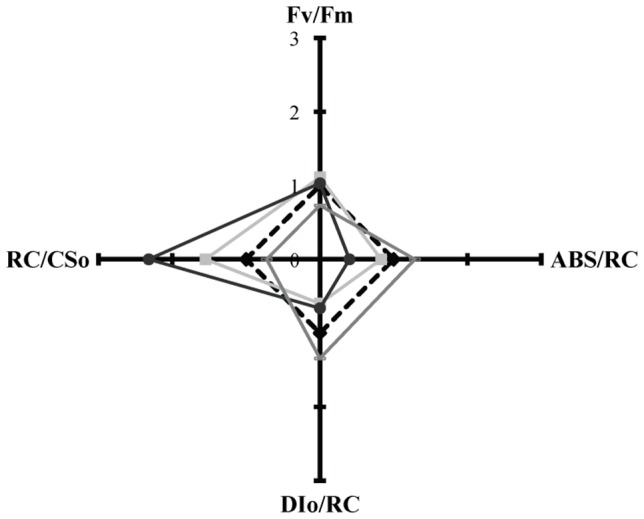
p-Cresol impact on the molecular structure and function of the microalgal photosynthetic apparatus. Changes in the molecular structure and function of the photosynthetic apparatus of *Scenedesmus obliquus* cultures, cultivated at different concentrations of 4-methylphenol (p-cresol) [*black diamond*: 0 mM, *grey square*: 0.15 mM, *dark grey circle*: 1.0 mM and *grey line*: 2.5 mM] for 24 hours. Fv/Fm : maximum photosynthetic efficiency, ABS/RC: functional antenna size, RC/CSo : active reaction centers density and DIo/RC: dissipation energy per active reaction center.

The changes induced in the molecular structure and function of the photosynthetic apparatus by the tested p-cresol concentrations, during the first incubation day (less secondary phenomena), were estimated using fluorescence induction measurements. According to the JIP-test parameters of control cultures (0 mM), the tested treatments were categorized into three distinct groups. The first group consists of concentrations 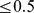 mM, the second one from 0.75 mM to 1.5 mM, while the last one consists of concentrations 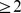 mM. A representative concentration of each group is shown in Fig. 2.

**Figure 3:**
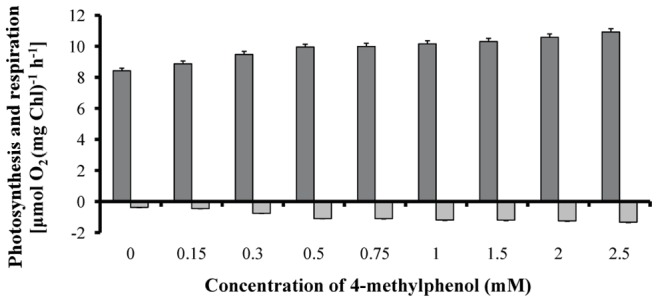
p-Cresol influence on the photosynthetic and respiratory rates. Maximal net photosynthetic (*dark grey*) and respiratory (*light grey*) rate of *Scenedesmus obliquus* cultures, cultivated in various 4-methylphenol (p-cresol) concentrations [Total chlorophylls in all experimental treatments was quite similar (0 mM p-cresol: 46.5+0.13 µg/mL; 0.15 mM p-cresol: 46.7±1.28 µg/mL; 0.3 mM p-cresol: 44.9±1.78 µg/mL; 0.5 mM p-cresol: 45.8±1.88 µg/mL; 0.75 mM p-cresol: 44.7±0.22 µg/mL; 1.0 mM p-cresol: 45.0±1.16 µg/mL; 1.5mM p-cresol: 45.1±0.72 µg/mL; 2.0mM p-cresol: 46.3±0.50 µg/mL; 2.5mM p-cresol: 43.4±1.01 µg/mL)].

The toxicity/stress effects of higher p-cresol concentrations (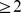 mM) led to the inactivation of the photosynthetic reaction centers (RC/CS_o_), the increase of the functional antenna size (ABS/RC) and, subsequently to the enhancement of the dissipation energy (DI_o_/RC) and the decrease of the photosynthetic efficiency (F_v_/F_m_) (Fig. 2). These responses are in agreement with observations on the molecular structure and function of the photosynthetic apparatus in microalgal abiotic stress conditions, such as high UV–B radiation [40], [41], and high salinity [42]. Conversely, there were no toxicity/stress effects on the microalgal photosynthetic apparatus for p-cresol concentrations 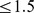 mM (Fig. 2). The changes induced in JIP-test parameters are usually associated with enhanced photochemical quenching of the absorbed energy and therefore enhanced photosynthetic rate. Polarographic measurements confirmed the above mentioned fluorescence data (Fig. 3).

**Figure 4:**
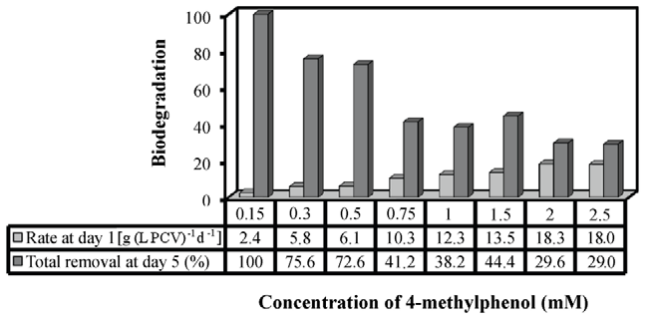
p-Cresol biodegradation by the microalga *Scenedesmus obliquus*. Biodegradation of increasing 4-methylphenol (p-cresol) concentration by the microalga *Scenedesmus obliquus*. (*light grey*): Biodegradation rate per PCV at day 1, (*dark grey*): total biodegradation per culture at day 5.

**Figure 5:**
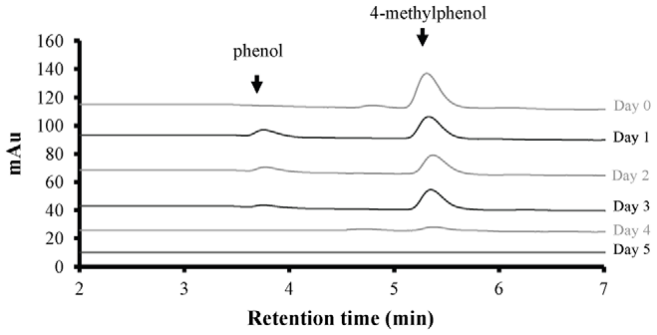
Biodegradation kinetics. Biodegradation kinetic of 0.15 mM 4-methylphenol (p-cresol) by the microalga *Scenedesmus obliquus* – HPLC profiles of the culture medium.

The p-cresol biodegradation rate after the first incubation day and the total p-cresol biodegradation after five incubation days, all expressed as percentage of the removal of the initial p-cresol amount are presented in Fig. 4. Higher p-cresol concentration led to higher biodegradation rate and lower total estimated biodegradability (Fig. 4). Particularly, 0.15 mM p-cresol was totally biodegraded within the five incubation days. The corresponding HPLC profiles are presented in Fig. 5.

The biodegradation data revealed p-cresol as an alternative carbon and energy source for carbon depleted *Scenedesmus* cultures. The ideal p-cresol concentration (100% biodegradability – Fig. 4 and 20% biomass increase of control – Fig. 1) was 0.15 mM. Concentrations of 0.3 and 0.5 mM p-cresol succeeded in terms of efficiency, with 75% biodegradability (Fig. 4) and 10% biomass increase compared to control cultures (Fig. 1). The positive influence of 0.15 – 0.5 mM p-cresol concentrations on carbon depleted *Scenedesmus* cultures was also obvious in the molecular structure and function of the photosynthetic apparatus (Fig. 2).

The combination of growth (Fig. 1), fluorescence (Fig. 2), polarographic (Fig. 3) and biodegradation data (Fig. 4) revealed a microalgal p-cresol biodegradation strategy. This strategy was regulated by the p-cresol concentration and consequently the energy requirements. Under carbon depletion the microalga needs carbon (as energy source) for growing. The biodegradation of p-cresol can be an alternative route for carbon assimilation. However, the initiation of the p-cresol bond fission demands extra energy. The initial carbon traces (see organism and growth conditions in Materials and Methods) and the continuous light illumination offered the energy (through photosynthesis) to start the biodegradation of p-cresol and use it as future carbon and energy source. Nevertheless, the different p-cresol concentrations altered the microalgal energy demands for the initiation of p-cresol biodegradation, due to different levels of initial culture toxicity.

**Figure 6:**
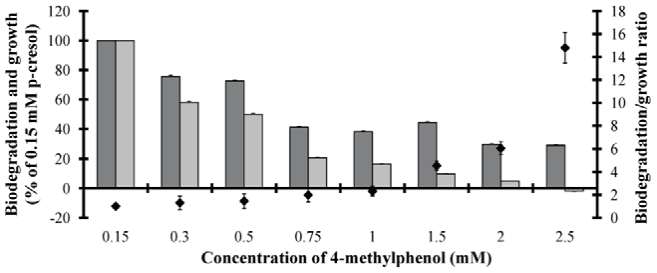
Biodegradation / microalgal growth ratio. Measurements of biodegradation, algal growth and the ratio biodegradation/growth in *Scenedesmus* cultures cultivated in different p-cresol concentrations. (*dark grey*): Biodegradation changes (% of 0.15 mM p-cresol treatment), (*light grey*): growth changes (% of 0.15 mM p-cresol treatment) and (*black diamond*): biodegradation/growth ratio.

It was observed that a higher p-cresol concentration resulted in a higher energy investment in biodegradation and a lower investment in growth (Fig. 6). This energy balance between biodegradation and growth is expressed by the biodegradation/growth ratio. This ratio was increased as the tested p-cresol concentration increased (Fig. 6). Further insights in p-cresol biodegradation suggested a two step biodegradation mechanism. The first step seems to be the split of the methyl group and the second one the fission of the intermediate produced phenol. Both methyl group and phenol can be further used as alternative carbon and energy sources.

The methyl group can be converted to methanol. The microalgal biomass increase associated with methanol formation and the changes induced in the photosynthetic apparatus [increased reaction center density (RC/CS_o_), photosynthetic efficiency (F_v_/F_m_) and maximal photosynthetic rate, decreased LHCII size (ABS/RC) and dissipation energy per active reaction center (DI_o_/RC)] – as observed in Figs. 1, 2 and 3 – have already been established in previous publications [43], [44], [45].

**Figure 7:**
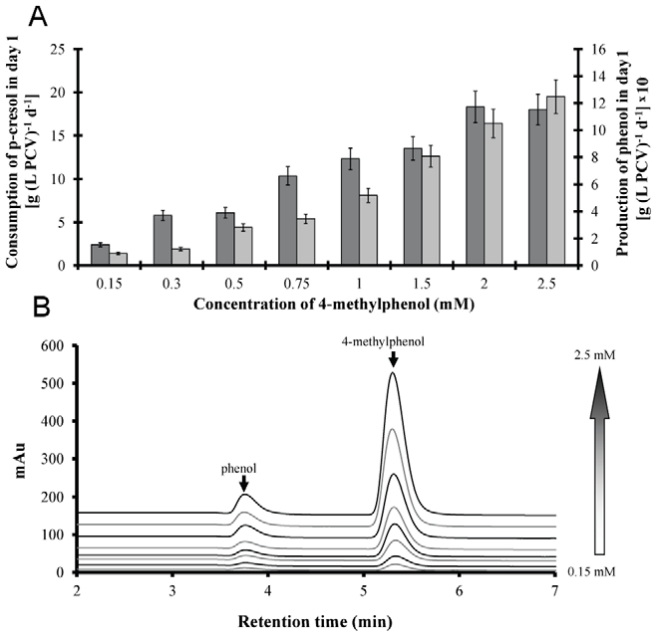
Consumption of p-cresol and phenol level in the microalgal cultures. Consumption of p-cresol and phenol level in *Scenedesmus obliquus* cultures cultivated in various p-cresol concentrations. (A) Quantification of p-cresol consumption (*dark grey*) and phenol level (*light grey*) after the first incubation day. (B) HPLC profiles of the culture medium after the first incubation day.

We previously published that very low methanol concentrations of 0.5% (v/v), induce an immense increase in biomass production in cultures of the unicellular green alga *Scenedesmus obliquus*. The effect is light-regulated and it mimics high-CO_2_ induced changes in the molecular structure and function of the photosynthetic apparatus. There is evidence that under high light conditions, methanol enhances the photochemical effectiveness of the absorbed energy through molecular changes in the LHCII; that is a decrease of the functional antenna-size per active reaction center. This means that the non-photochemical quenching (NPQ) is minimized and thereby the overall dissipation energy [44]. The HPLC profiles (Fig. 5) confirmed the above two steps p-cresol biodegradation mechanism. It was observed that higher p-cresol removal led to increasingly higher phenol concentrations (Figs. 7A and 7B). Recent results showed that methanol [44], [45] and phenol [22] can be used as alternative microalgal carbon/energy source.

**Figure 8:**
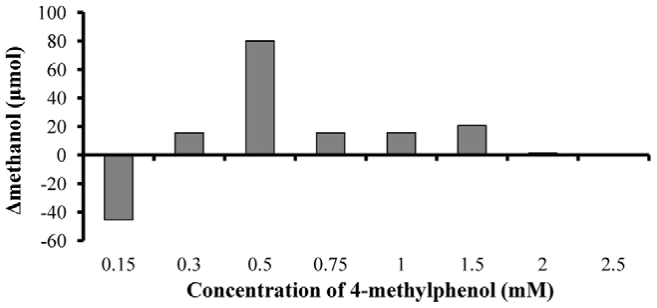
p-Cresol biodegradation and methanol formation. Changes of the methanol amount after 3 incubation days in all different p-cresol treatments.

**Figure 9:**
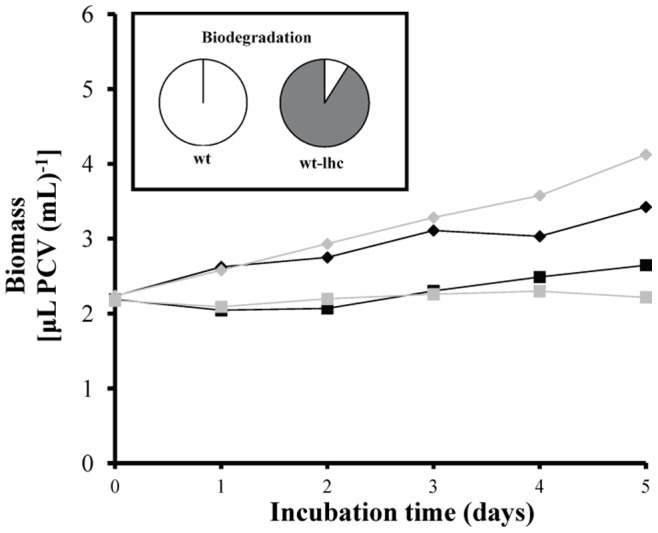
The necessity of LHCII for the biodegradation of p-cresol by the microalgae. Comparison of *Scenedesmus obliquus* wild type and wt-lhc mutant cultures incubated with 0.15 mM p-cresol for 5 days. Growth of *Scenedesmus obliquus* wt (*black diamond*: control, *grey diamond*: 0.15 mM p-cresol) and wt-lhc (*black square*: control, *grey square*: 0.15 mM p-cresol) cultures in terms of PCV. (insert): Biodegradation of 0.15 mM p-cresol by the wt and the wt-lhc mutant of *Scenedesmus obliquus* after five incubation days. (*white*): p-cresol removed and (*dark grey*): p-cresol remaining.

The possible continuous methanol production and consumption in the culture medium didn’t allow the quantification of the produced methanol in terms of absolute values. Although it was a dynamic process, we measured the actual methanol level in the culture medium in all different treatments as changes of the methanol amount in the 3^rd^ incubation day (Fig. 8). The results supported our initial hypothesis. We measured the total methanol amount at the onset of the incubation period (p-cresol diluted in methanol) and compared them with the corresponding methanol amount after the 3^rd^ incubation day. Treatments with p-cresol concentrations lower than 2 mM increased their methanol amounts compared to the onset of the experiment, except for 0.15 mM (Fig. 8). The above exception possibly was attributed to the consumption of methanol for higher growth rate (see the ratio biodegradation/growth rate in Fig. 6). Treatments with 0.5 mM p-cresol showed the highest amount of methanol (Fig. 8) because they increased their biodegradation rate (Fig. 4) without increasing in parallel their growth rate (Fig. 1). In a previous work it has been proven that the absence of LHCII (wt-lhc mutant of *Scenedesmus obliquus* – see Organism and growth conditions in Materials and Methods) blocked the methanol effect on the photosynthetic apparatus and subsequently the microalgal biomass increase [44]. For this reason, the same mutant was used in order to check the possible effect of methanol production (and necessity of functional LHCII) in p-cresol biodegradation. The wt-lhc mutant was not expected to biodegrade p-cresol probably because it could not metabolize the produced methanol (product of the first biodegradation step). The biodegradation measurements confirmed the above hypothesis. The total biodegradability of 0.15 mM p-cresol was only 8.9 % for the mutant (Fig. 9) instead of 100% (Fig. 4) of the wild type. As a result, there was no difference in the mutants’ growth rate in the presence of p-cresol (Fig. 9) in contrast to the 20% biomass increase of the wild type (Fig. 1).

### Conclusions

The main conclusions of the present contribution can be summarized in the following points:

- Cultures from the unicellular green alga *Scenedesmus obliquus* biodegrade the toxic p-cresol (4-methylphenol) and use it as alternative carbon/energy source.
- The biodegradation procedure of p-cresol seems to be a two-step process. HPLC analyses indicate that the split of the methyl group that is possibly converted to methanol (increasing methanol concentration was measured in the growth medium), leading, according to our previous work [44], to changes in the molecular structure and function of the photosynthetic apparatus and therefore to microalgal biomass increase. The second step is the fission of the intermediately produced phenol.
- The first biodegradation step seems to be the most decisive for the effectiveness of the process, because methanol offers energy for the further biodegradation reactions. The absence of LHCII from the *Scenedesmus* mutant wt-lhc stopped the methanol effect and also significantly reduced the p-cresol biodegradarion [the total biodegradability of 0.15 mM p-cresol was only 8.9 % for the mutant (Fig. 9) instead of 100% (Fig. 4) of the wild type].
- A higher p-cresol concentration results in a higher p-cresol biodegradation rate and a lower total p-cresol biodegradability (the amount of the biodegraded p-cresol in percentage of the initial concentration).
- The present contribution deals with an energy distribution between microalgal growth and p-cresol biodegradation, activated by p-cresol concentration. The findings support the importance of the bioenergetic balance, via cellular energy management, in the biodegradation of p-cresol by the unicellular green alga *Scenedesmus obliquus*. The simultaneously biomass increase with the detoxification of a toxic phenolic compound (p-cresol) could be a significant biotechnological aspect for further applications.
